# Immune Aspects of Female Infertility

**DOI:** 10.22074/ijfs.2016.4762

**Published:** 2016-04-05

**Authors:** Andrea Brazdova, Helene Senechal, Gabriel Peltre, Pascal Poncet

**Affiliations:** 1 Department of Biochemistry, Allergy and Environment, Armand-Trousseau Hospital, Paris, France; 2 Department of Infection and Epidemiology, Pasteur Institute, Paris, France

**Keywords:** Immunoglobulins, Infertility, Sperm

## Abstract

Immune infertility, in terms of reproductive failure, has become a serious health issue involving approximately 1 out of 5 couples at reproductive age. Semen that is defined as a complex fluid containing sperm, cellular vesicles and other cells and components, could sensitize the female genital tract. The immune rejection of male semen in the female reproductive tract is explained as the failure of natural tolerance leading to local and/or systemic immune response. Present active immune mechanism may induce high levels of anti-seminal/sperm antibodies. It has already been proven that iso-immunization is associated with infertility. Comprehensive studies with regards to the identification of antibody-targets and the determination of specific antibody class contribute to the development of effective immuno-therapy and, on the other hand, potential immuno-contraception, and then of course to complex patient diagnosis. This review summarizes the aspects of female immune infertility.

## Introduction

The World Health Organization declares infertility as a disease of the reproductive system defined by the failure to achieve a clinical pregnancy after 12 months or more of regular unprotected sexual intercourse. Infertility has been reported to be one of the most prevalent chronic health disorders regardless of age (1,2). The decreased fecundity is associated with other health issues (severe avitaminosis, severe renal impairment, cancer and cachexia due to malnutrition or tumor), age, lifestyle and environment. The male partner accounts for the infertility 40% of the time, 40% from the female partner as well and 20% shared by both the man and the woman. The factors involve congenital, hormonal, morphological and immunological disorders (3). The main disorders involved in infertility include pathologic spermiogram, ovulation problems/anovulation, tubal diseases, pelvic adhesion/endometriosis, cervical factors and idiopathic reason usually qualified as the so-called unexplained infertility (UI) (4,6). 

UI is diagnosed in a couple when the standard investigations including the semen analyses, test of ovulation and tubal potency do not provide specific results or do not detect any abnormality. Several reports (4,5,7) suggested that the diagnosis of UI is subjective and often misdiagnosed for endometriosis, tubal infertility, premature ovarian ageing and immune infertility. The prevalence of UI reaches up to 30% of infertile couples with regards to standard investigation. Severe endometriosis affects the fecundity potential. Mild endometriosis is not, however, associated with infertility in the absence of secondary organic disruption. It has been reported that approximately 20% of infertile females suffer from tubal disease, either distal or peritubal (2,4). Follicle number is genetically dependent. Female subfertility caused by poor ovarian reserve is declared when the remaining follicle amount represents a fraction of the original value (8). In some women, the so-called poor ovarian response has been noticed when the ageing ovary produces fewer follicles, follicles grow poorly and follicular atresia occurs (5). Molecular and cellular endometrial deficiency resulting in an implantation failure can be related to UI since the natural immunosuppression does not prevent maternal immune rejection. T regulatory (Treg) cells are believed to protect the fetus from an immune attack. Treg cells function in immune tolerance exhibiting the immuno-suppressive activity. A factor of spontaneous abortion is displayed in the case of a lower number of of CD4^+^CD25^+^ Foxp3^+^ Treg cells that is, under normal conditions, elevated in the first trimester of physiological pregnancies(9,10). UI is not necessarily linked to Treg differentiation, thus to immune suppression failure, but also to its recruitment into the implantation site. This fact is caused by the reduced expression and insufficient function of lymphocyte and chemotactic agents present in the uterus. Since Treg differentiation is regulated by transforming growth factor beta (TGFβ), idiopathic infertility may be related to a reduced availability of this factor. The lack of TGFβ results in insufficient Treg induction. Diminished CD4^+^CD25^+^ Treg population, the lower expression of Foxp3 and the failure of lymphocyte adherence and chemotaxis seem to play, however, a role in primary cause of UI (11). 

Immune/immunological infertility is diagnosed when spontaneously produced antibodies bind to the antigens occurring on either the male or female gametocytes. In particular, antibodies bind to seminal proteins or structures present on the sperm or oocyte. So far, anti-sperm antibodies (ASA) have been observed more frequently than anti-oocyte antibodies (12). 

## Antibody formation

After an exposure to an antigenic agent, the level of immunoglobulin M (IgM) antibodies is supposed to be dominant at the early phase of a primary immune response. In response to some allergens, IgE antibodies may be prevalent in genetically predisposed individuals. The switch into IgG and IgA antibodies is induced at the late phase of primary immune response or after repeated exposure to the same antigen (13-15). When chronically exposed to the antigens, IgG_1_ and IgG_4_ become the predominantly produced subclasses of IgG isotype. IgG_4_ is a unique antibody unable to activate the classical complement pathway and is then known as an anti-inflammatory Ig and a blocking antibody towards IgE antibodies, depending on the antigenic model. It remains unclear whether IgG_4_ is a protective or pathogenic antibody (6, 16, 17). Schroeder and Cavacini speculated that IgG_1_ and IgG_3_ antibodies are generally induced in response to protein antigens whereas IgG_2_ and IgG_4_ to polysaccharide antigens (18). Other studies related to antibody distribution neither refute nor endorse this hypothesis (6, 19). We have reported IgG_1_/ IgG_4_ predominance in anti-seminal antibodies and IgG_4_/IgG_1_ predominance in ASA. We have also proposed the distribution of seminal/sperm-specific antibody isotypes showing that immunoglobulins E, M, A_1, 2_, G_3_ are not significantly involved in pathophysiological female sensitization. Specific IgG_4_ appears to be mainly produced together with specific IgG_1_ (20).

## Anti-sperm antibodies

The sperm antigenicity concerning the animal kingdom was first described by Landsteiner, Metchnikov and Metalnikova in 1899 as sperm toxins. In 1932, Baskin observed circulating antibodies against sperm and in 1954, Rümke observed and described the first type of ASA. They have cytotoxic, immobilizing and agglutinating functions. ASA are detectable on the systemic (blood and lymph) as well as the local level [seminal fluid (SF), cervical-vaginal mucus]. In general, the IgG isotype of ASA is mainly related to the blood circulation and IgA isotype to mucosal immunity in women. In men, IgG and IgA fractions are the most prevalent in SF, while IgG and IgM isotypes in serum (6,21). 

Semen has a very heterogeneous antigenic content. Since sperm has auto-antigenic (auto-immunization) as well as iso-antigenic (iso-immunization) potential, it is able to induce the production of sperm-reactive T-cells in men as well as in women, thus is opsonized and then targeted by the leukocytes (sperm-cytotoxic effect) (22,24). It is not a single ASA that influences fertility but more likely multiple ASA causing infertility. Furthermore, it has been postulated that antibodies against a single sperm antigen cannot cause infertility. It has also been reported that not all ASA, either produced in women or men, affect the fertility potential since the cognate antigen is not necessarily involved in the fertilization process (6,23,26). 

A highly heterogeneous sperm antigenic content could be modified during maturation and ejaculation based on antigen sequestration. Newly expressed antigens could then be in contact with any immunocompetent cells, e.g., a sperm membraneincorporated fibronectin exhibits changes in regional antigenic expression during sperm maturation, whereas secreted fibronectin is a product of male accessory sex glands and can be attached to sperm tail during ejaculation (12,27). Considering gastrointestinal exposure, ASA formation may be operative (21). 

In men, sperm germ cells are protected in the testis from an auto-immune attack by the bloodtestis barrier. When the barrier is disrupted, autoantibodies are produced and are then detectable in blood serum, seminal plasma or directly attached to the sperm surface membrane (28). An increased risk of ASA formation may follow the congenital absence of reproductive tract components. ASA are mostly associated with genital inflammation/ infection (e.g., orchitis), epididymis trauma, genital surgery, cryptorchidism and varicocele (27). The theory of auto-immune disease was supported by proving that ASA formation is related to certain human leukocyte antigen classes (29). 

In women, the failure of natural tolerance may lead to sensitivity resulting in sperm elimination. ASA affect fertility potential through various pre/ post-fertilization processes, such as sperm agglutination and motility, cervix mucus penetration, capacitation, acrosome reaction, zona pellucida (ZP) binding and penetration, oolemma binding, spermoocyte fusion and embryo implantation (30,31). The active local immuno-regulatory mechanism is based on vaginal and cervical tissues having an active and sensitive mucosal immune system, by which the fertility potential is affected. This explains the rather high percentage of infertile women with the local reactions leading to inflammation as well as with high levels of serum anti-semen antibodies. Furthermore, ASA-coated sperm may be more vulnerable to phagocytosis in the female reproductive tract (28). Serum ASA are related to the long-term exposure of female to sperm and then to seminal deficiency in immuno-suppressive factors (32,33). 

Nevertheless, there is the evidence of ASA occurrence in fertile women and men. Some fertile individuals are positive in serum sperm agglutinins. It has been suggested that these ASA are not clinically significant. It is a physiological effect without a pathologic background as they do not inhibit the fertilization process either *in vitro* or *in vivo* (12,23). In this case, they may be considered as the so-called natural ASA (34). They are produced by auto-reactive B cells in men that were stimulated to grow. Furthermore, natural autoantibodies may be more poly-reactive antibodies, hypothetically help remove senescent molecules and cells, and participate in immune auto-treatment of cancer (35,36). The poly-reactive character may play a part in cytotoxic reaction at early fertilization associated with infertility. 

## Role of seminal fluid in female immune infertility

SF represents a part of the semen containing a range of organic/inorganic substances ( e.g., neutral α-glucosidase, hyaluronidase, carnitine, glycerolphosphocholine, fructose, prostaglandins (PGs), citrate, zinc, selenium) that are necessary for the physiological metabolism of sperm. The seminal complex mixture of secretions originates in the testis, epididymis and accessory glands including the prostate, seminal vesicles and Cowper’s gland. It also acts as a nutritive, transport and buffering medium of pH=7.35-7.5 that defines the main SF functions: sperm protection from the acidic environment of the vagina, metabolic support, liquefaction and clot formation. SF composition is similar to blood plasma; however, it differs in saccharide content (37,39). 

Prostate specific antigen (PSA), prostatic acid phosphatase (PAP) and prostate-specific protein-94 belonging to the prostate secretion are in direct contact with sperm and thus may be the first to confront the cervical tissues. PSA is a 33 kDa member of the glandular kallikrein subfamily of serine proteases participating in the liquefaction of the seminal coagulum. Its activity is strongly inhibited by zinc ions (40,42). Serum PSA is a commonly used marker of prostate cancer (43,45). PAP, a member of the histidine acid phosphatase family, is a non-specific tyrosine phosphatase that dephosphorylates macromolecules and inactivates lysophosphatidic acid in SF (46,47). Seminal components, e.g., heparin (48) or zinc-2-glycoprotein (ZAG) (49), bind to the acrosomal sperm head region protect sperm and are then carried together into the higher female genital tract. SF plays an important role in moving the sperm into the female reproductive tract due to its high content of TGFβ and PGE, both of which inhibit the function of natural killer (NK) cells and neutrophils that are recruited into the superficial epithelial layers of the cervical tissues. TGFβ is synthesized in the prostate and is testosterone-dependent. This glycoprotein belongs to cell-secreted molecules and occurs in 75% in the latent form in SF. It is further activated in the female reproductive tract by either the enzymes of male/female origin, acidic vaginal pH or through conformational change after an interaction with epithelial cells. The remaining proportion of TGFβ, 25%, exists in an active form (50,51). TGFβ acting may result in the immune tolerance of seminal antigens. A divergent member of this family is growth/differentiation factor 15 (GDF 15), which is highly abundant in SF. GDF 15 has anti-tumorigenic activity, serves as a cancer marker and is likely to promote a pro-inflammatory immune response. High level of GDF 15 in female serum corresponds to spontaneous abortion as it is expressed in the placenta. It has been suggested that due to the presence of seminal antigens on a fetus, TGFβ facilitates the female immune tolerance to the fetus (52,53). 

Some seminal constituents, such as cathepsin D, are able to degrade proteins vaginally exposed that may be involved in antibody formation related to immune infertility (54). Seminal ZAG has been reported as a novel adipokine playing a significant role in fertilization, lipid mobilization, and peptide/antigen/ligand binding. ZAG may participate in the expression of female immune response since the fold is similar to major histocompatibility complex (MHC) molecules, in particular MHC I, on the antigen-presenting cells. ZAG has been proven to be an IgG-binding protein related to a pathophysiological iso-immunization. This protein belongs to immunoglobulin gene family and may have a protective role by blocking the elicited female anti-semen antibodies (6,31,49). SF includes a repertoire of signaling molecules interacting with the epithelium in the female reproductive tract. SF may modulate the chemotactic and phagocytic responses of the female reproductive tract. Phagocytes serve to filter out morphologically abnormal sperm. Sperm selection is based on morphological or antigenic structures. Mainly, the immune modulating properties are mediated by the PGs of the E series, complement inhibitors, cytokines and proteins capable of binding IgG antibodies (55,56). Local reactions may lead to an inflammation. However, SF has a built-in mechanism preventing an immunological sensitization of the female against sperm as well as seminal structures. This protective system exists due to the presence of immune inhibitors originating in the male sex accessory glands (52,57). 

SF has been suggested to be the modulator of sperm-induced inflammation that leads to sperm elimination from the female genital tract. Antibody fraction interacting with seminal antigen targets most of the seminal proteins adsorbed on sperm. However, SF induces the recruitment of macrophages and dendritic cells into cervical and endometrial tissues (58). SF elicits endometrial changes by inducing pro-inflammatory cytokines and cyclooxygenase-2. Their presence leads to macrophage and dendritic cell recruitment into the female reproductive tract. Seminal components activate the income of neutrophils into the endometrial stroma (24,52,59). However, it has been reported that the influx of neutrophils is higher and faster when the washed sperm inseminated (60). This fact demonstrates the protective and signaling activity of SF. The immuno-suppressive activity prevents the iso-immunization of the female reproductive tract and suppresses cell-mediated cytotoxicity (61). Seminal prostaglandin D2 is known for its immuno-suppressive effect, by which ASA formation is prevented in the female genital tract. The immuno-modulating properties are mediated by PGE, complement inhibitors, cytokines and proteins capable of binding the Fc region of IgG. These IgG-binding proteins are Fcγ receptor-like soluble proteins. 

In general, seminal antibody-binding proteins contribute to sperm protection against immunemediated damage by enabling successful sperm passage in the female reproductive tract and by blocking an interaction with immune effectors such as prolactin-inducible protein, which is a secretory glycoprotein located in seminal vesicles, binds to immunoglobulin G via its Fc fragment. It may therefore be involved in immune regulation by trapping ASA and neutralizing them (62,63). Particular deficiencies in seminal factors may lead to higher antibody production in infertile women (64). 

SF has already been considered to be linked to the IgE-mediated rare reaction to semen (65). This rare phenomenon was first reported in 1945 (66). Human seminal plasma allergy (HSPA), the so-called hypersensitivity to semen, is defined by local and/or systemic symptoms after exposure to SF. The symptoms occur immediately after contact with semen or even within several hours after intercourse. The local symptoms include vulvar/ vaginal itching, burning, redness and swelling. Local reaction can appear on any semen contact site and can be misdiagnosed as chronic vulvo-vaginitis caused by bacteria, yeasts, viruses and other parasites. Systemic symptoms include generalized urticaria, angioedema (face, tongue, lips, throat), dyspnea, wheezing, cough, chest tightness, rhinorrhea, nausea, vomiting, diarrhea. Generalized malaise may result in an anaphylactic shock, which is a life-threatening reaction. The symptoms can manifest after the first time intercourse in up to 50% of cases. Response mediated by IgE antibodies is then the most common mechanism. It has been suggested that female patients experiencing any allergic symptoms after/during the first time intercourse might be sensitive to other antigens/ allergens that cross-react with SF. IgE cross-reactivity has already been proven among proteins from dog epithelium and PSA (67). Patients diagnosed with HSPA have difficulties conceiving but infertility has not been demonstrated, so far (65,68,69). 

## Auto-immune aspects in infertility

Auto-immune phenomena have already been associated with increased prevalence of female immune infertility. This fact concerns anti-phospholipid, anti-nuclear, anti-thyroid, anti-annexin V, anti-prothrombin, anti-laminin, anti-ZP antibody formation, the high level of NK cells as the risk factors but not as those pathognomonic (4). 

ZP, as the protective layer, is composed of glycoproteins. It represents a broad antigenic content. Antibodies against ZP prevent sperm from penetrating it. Anti-ZP autoantibody concentration can be elevated if ZP shape is abnormal (deformed, thickened, thinned). These antibodies interfere with the implantation process since ZP protects a fertilized oocyte up to the 7^th^ day after fertilization, up to embryo hatching. During this time the ZP is thickened (15-17 μm). ZP-specific antibodies are detectable in follicular and peritoneal fluid, and cervical mucus in IgG, IgA and IgM isotypes (21). 

Anti-phospholipid antibodies (APA) have been associated with e.g. miscarriage, intrauterine fetal death, and placental thrombosis since the time of their discovery by Wasserman in 1906. These components of the female immune system are autoantibodies directed in particular against β2glycoprotein, phosphatidylserine, phosphatidylinositol, phosphatidylethanolamine, annexin V and cardiolipin. APA are mostly produced in IgG fractions accompanied by IgA and IgM. Phosphatidylserine-specific APA cause fetus hypotrophy as a consequence of placental vascular damage, against which the maternal immune system produces anticoagulating factors (70). The risk of spontaneous abortion increases with the presence of anti-coagulating antibodies. Antibodies specific to annexin V and placental anti-coagulating protein are also related to reproductive failure and detectable in 5-6% of women diagnosed with pregnancy loss, 8-10% of women after unsuccessful *in vitro* fertilization, 1% of not pregnant and healthy women, and 0% of pregnant women without a pathophysiologic aspect. Complex complication is called anti-phospholipid syndrome also known as Hughes syndrome. It may cause hyper-coagulation leading to rapid organ failure (6,21,70). 

Endometrium-specific antibodies are, inter alia, associated with polycystic ovary syndrome (PCOS) that is mainly classified as an endocrine genetic disorder. PCOS is known as SteinLeventhal syndrome first described in 1935. It is characterized by enlarged ovaries caused by cysts, irregular ovulation, irregular or no menstruation, and increased androgen levels. With regards to androgen levels, PCOS is associated with hirsutism. On the other hand, it is associated with obesity, type 2 diabetes and high cholesterol levels. Women suffering from PCOS have usually problems with conceiving (21,71). 

Pregnancy is also complicated by endometriosis, a serious gynecological complication affecting up to 10% of women of reproductive age. Twentyfive % of women diagnosed with endometriosis are infertile. Peritoneal endometriosis is characterized by retrograde menstruation causing secondary inflammation. Factors typical for such a condition are high level of autoantibodies, presence of T-lymphocytes in peritoneal fluid, and elevated level of NK cells (72,73). 

## Mucosal immunity of the female genital tract

The mucosal immune system operates on a local level and is represented by lymphoid tissues in mucosae and external secretory glands. It limits the access of environmental antigens by which the fertility potential is significantly regulated as well. It restricts and/or prevents the penetration in the systemic compartment. The female genital tissues and secretion (vaginal washes and cervical mucus) provide the protection that differs from systemic reaction by the cell types involved and by their products, the antibodies. However, it is the initial antigen exposure to mucosae that leads to the systemic T cell hypo-responsiveness (74,75). 

Mucosal immunity in the female genital tract is influenced by the level of antibodies, cytokines and hormones. Humoral defense displayed in mucosal tissue surface provides the antibodies of the IgG, IgA and IgM isotypes. IgG, IgA and IgM levels are dependent on the menstrual cycle and are influenced by hormones. IgA and IgG reach their maximum concentrations before ovulation, which is linked to the increased level of interleukin 1 component β. In particular, estrogen causes a higher expression of secretory IgA (S-IgA), thus its selective transport is increased. This way of regulation is responsible for antibody-isotype distribution including their properties, the transport of immunoglobulin- containing cells, antigen-presenting cells, in addition to CD4^+^ and CD8^+^ cells in the vagina, uterus and fallopian tubes (76). In addition, it has been shown (77) that oral contraception influences IgA as well as IgG levels in the cervical mucus. It is almost one third higher than in the cervical mucus of naturally cycling women. The vaginal washes of women on oral contraception display an elevated level of IgG in comparison to IgA. Several observations showed (76, 78) that the concentration decreases in this manner: IgG>IgA>IgM. This ratio is related to the presence of IgG/A/Mproducing cells. The uterine endocervix contains the highest amount of IgGand IgA-secreting cells compared to the ectocervix, fallopian tubes and vagina (76, 79). Cervical mucus contains higher levels of IgG than IgA, both of which are locally produced. On the contrary, women on oral contraception have IgA as the predominant antibody present in cervical mucus. Among the three mentioned isotypes, IgM is the less efficiently transported antibody. The mucosal IgA antibodies are selectively transported to an external secretion based on a receptor-associated mechanism. The distribution of IgG subclasses in mucosal secretions displays a plasma proportion. IgD occurs rarely or in very low concentrations in external mucosae. The level of IgE depends on the genetic predisposition to develop allergies and then on the allergenic nature of the presented antigen (74). Despite the low IgA affinity, the avidity is high regarding the multibinding sites. Environmental antigens are usually degraded by proteolytic enzymes. IgA itself is resistant to the enzymes of proteolytic character. It has been suggested that a certain amount of not eliminated antigens circulates in the complex with IgA, which further activates the systemic immune response. It is less probable that antigens entering, at first, the mucosal tissue could circulate on itself (75, 78, 80). IgA is a multivalent antibody existing in two subclasses, IgA_1_ and IgA_2_. IgA has an antiinflammatory activity proved by the inhibition of complement activation and by a diminishing effect on NK cells. These properties may avoid an earlyprecise diagnosis as an inflammatory marker and may not be detected. In cervical mucus as well as a vaginal wash, the IgA_1_ concentration is equal to IgA_2_. S-IgA is locally produced by sub-epithelial plasma cells. Most of the time, it is a polymeric molecule, which corresponds to IgA_2_ since IgA_1_ is rather monomeric. It has been suggested that cervical mucus contains approximately 80% of the polymeric form and the vaginal wash contains approximately 50% (74, 76). Eosinophils that cover mucosal surfaces can be degranulated by IgA antibodies. This pathology is observed when natural immune tolerance is disrupted. Further reactions may evoke an allergic reaction to the presented antigen, such as seminal and/or sperm component. Semen rejection at the level of mucosal immunity may not be reflected at the systemic level (74). The protective role depends on an antibody-dependent cell-mediated cytotoxicity, opsonization, the activation of innate humoral factors, removal and further elimination of already formed immune complexes within epithelial cells and lamina propria. IgA is able to diminish absorption of an entire antigen as well as a part of it on mucosal tissues. In comparison with IgG, which after antigen-recognition activates complement resulting in inflammation, IgA acts as an inhibitor or directly avoids the adherence of antigen (81). S-IgA in a complex with an antigen is not able to efficiently activate the complement pathway. IgA antibodies have been reported to be a part of the natural antibody pool showing the characteristics of polyreactivity and hypothesized to act as the first barrier defense (82).

The uterine cervix participates in the local immune reaction by the presence of immunoglobulin-producing cells in a complex mixture known as cervical mucus/fluid/plasma. The fluid located in and around the cervix. Cervical mucus is composed mostly of water, up to 90%, depending on the menstrual cycle. Its composition is based on a glycoprotein web filled by mucus rich in immunocompetent proteins, electrolytes (calcium, sodium and potassium), simple sugars such as fructose and glucose, amino acids, C3 and C4 complement components, Th1 and Th2 cytokines, PGE, and trace elements (zinc, copper, iron, manganese, selenium). The misbalance in its content is frequently associated with immune infertility and spontaneous abortion (83,86). The value of pH is alkaline especially at ovulation in order to allow sperm survival by the elevated level of water and electrolytes. After menstruation, cervical mucus becomes rather acidic. Acidic pH is characteristic for vaginal mucus as well (87). 

The basic role of cervical mucus consists in a barrier blocking uterus entrance. It is connected to “stick and thick” properties and acts as a natural lubricant because of its glycerol content. Its amount is not hormone-dependent. The mucus functions also as a transport and nourishing medium for sperm by being less concentrated, transparent with a lower amount of immuno-competent agents and high fructose level, which is essential for efficient sperm metabolism. The sugar level is progesterone dependent (21,84,85). On the other hand, the cervix always acts as a reservoir for sperm after sexual intercourse. Regarding the iso-immunization within the entire menstrual cycle, cervical mucus contains the antibodies directed to sperm. Their level is then crucial for sperm-cervical mucus penetration and following fertilization. ASA-positive female patients have been commonly diagnosed with immune infertility. An iso-immunization rate is observed by a local ASA level which determines the appropriate treatment (84,85,88). It has been shown (86) that ASA present in cervical mucus are of an agglutinating character. These locally produced ASA do not differ from those systemically produced, thus they affect sperm capacitation, acrosome reaction and may interfere with ZP penetration as well as embryo implantation. The peak of ASA in IgA and IgG fraction is reached at the luteal and follicular phases of the menstrual cycle. In contrast, their level is lowest at ovulation. (77). 

## Conclusion

Infertility has been defined as reproductive failure and recognized as a disease. Idiopathic infertility correlates with certain immune aspects such as natural tolerance, in addition to the levels of immunoglobulins and specific antibodies in local and systemic secretions. Sperm displays a very heterogeneous antigenic content and has highly autoas well as iso-antigenic potential. SF, a protective and nutritive sperm medium, is the first contact with the local immune system of the female genital tract, thus representing the potential antibody-targets. Mucosal immunity in the female genital tract is influenced by the level of natural and specific antibodies, cytokines and hormones. Seminal components also bind to the acrosomal sperm to protect it and are then carried together with it into the higher female genital tract. Iso-immunization has been associated with female immune infertility. The thorough comprehension of this pathophysiological process consists of the determination of antibody isotype mostly involved in antigen targeting; and on the other hand, consists of the characterization and identification of semen antibody-binding proteins. In particular, early determination of serum seminal/ sperm-specific immunoglobulin G subclasses may make patient profiling more precise and complete the information for diagnosis. Furthermore, based on our studies, anti-seminal/sperm IgG_1_ and IgG_4_ could be of interest for further therapy targets. The identification of uniform autoand iso-immunization markers would contribute to a comprehensive, detailed patient diagnosis. 
